# Patient and family engagement in patient safety in the Eastern Mediterranean Region: a scoping review

**DOI:** 10.1186/s12913-024-11198-3

**Published:** 2024-06-25

**Authors:** Zhaleh Abdi, Hamid Ravaghi, Samaneh Sarkhosh, Hamideh Nafar, Sedigheh Khani, Mondher Letaief

**Affiliations:** 1 National Institute of Health Research (NIHR), Tehran University of Medical Sciences (TUMS), Tehran, Iran; 2 Department of Universal Health Coverage/Health Systems (UHS), World Health Organization, Regional Office for the Eastern Mediterranean, Cairo, Egypt; 3 School of Health Management and Information Sciences, Iran University of Medical Sciences (IUMS), Tehran, Iran; 4 National Library and Archives of Iran, Tehran, Iran

**Keywords:** Patient safety, Patient involvement, Patient engagement, Eastern Mediterranean Region, Scoping review

## Abstract

**Background:**

Patients can play a key role in delivering safe care by becoming actively involved in their health care. This study aimed at reviewing the literature for evidence of patients’ and families’ engagement in patient safety in the Eastern Mediterranean Region (EMR).

**Methods:**

We conducted a scoping review of the literature published in English using PubMed, Medline, CINAHL, Scopus, ISI Web of Science, and PsycINFO until June 2023.

**Results:**

A total of 9019 studies were screened, with 22 meeting the inclusion criteria. Our review found few published studies of patient and family engagement in patient safety research in the EMR. Thirteen studies explored the attitudes, perceptions, and/or experiences / preferences of patients, families, and healthcare providers (HCPs) regarding patient engagement in patient safety. Nine publications reported patient involvement in patient safety activities at varying levels. Three categories of factors were identified that may affect patient involvement: patient-related (e.g., lack of awareness on their role in preventing harms, unwillingness to challenge HCPs’ authority, and cultural barriers); HCP-related (e.g., negative attitudes towards patient engagement, poor patient-provider communication, and high workload); and healthcare setting-related (e.g., lack of relevant policies and guidelines, lack of training for patients, and HCPs, and lack of patient-centered approach).

**Conclusion:**

This review highlighted limitations in the current literature on patient and family engagement in patient safety in the EMR, including both the depth of evidence and clarity of concepts. Further research is needed to explore how to actively involve patients and their families, as well as to determine whether such involvement translates into improved safety in practice.

**Supplementary Information:**

The online version contains supplementary material available at 10.1186/s12913-024-11198-3.

## Background

Patient safety is recognized as a serious public health concern in both developing and developed countries [1]. Despite substantial efforts over the past two decades, patient safety incidents remain a leading cause of disability and death, contributing significantly to increased healthcare costs worldwide [2]. Patient safety is fundamental to delivering high-quality essential health services and is core to achieving universal health coverage (UHC) and optimal healthcare delivery worldwide [3].

Engaging patients in promoting safety and reducing adverse events has become an international policy priority [4, 5]. The World Health Organization (WHO) has recognized the necessity of empowering patients, families, and communities to ensure their sustained and effective engagement at all levels of health care as a core strategy to make healthcare services safer [6]. The World Alliance for Patient Safety was established by the WHO with the goal of coordinating and expediting global initiatives to enhance patient safety [7]. Patient and community engagement was one of the six original core focuses of the World Alliance for Patient Safety [3]. Despite the emphasis on involving patients in promoting safety and reducing adverse events, insufficient progress has been made worldwide in this area [8, 9].

Evidence suggests that most patients are willing and able to participate and engage in their safety, and their participation has been associated with enhancing patient safety [10, 11]. When patients and their family members participate in the process of care, they can provide a safety net by compliance with prescriptions and self-management, observing and checking care processes, alerting care teams on concerning symptoms, speaking up and raising concerns, identifying and reporting possible treatment complications and adverse drug events, checking the accuracy of medical records, and practicing in targeted interventions to promote safety [12]. During the COVID-19 pandemic, the lower level of family participation in the care processes, due to restrictions prohibited them from visiting patients, was linked to a rise in the number of adverse events experienced by patients [13].

Patient engagement efforts in quality and safety span a range of healthcare services, including community primary care (such as ambulatory care settings and home-based care), secondary healthcare, and tertiary specialized care [14]. This engagement occurs along a continuum, ranging from consultation (i.e., one-way communication through receiving information in the context of their own care) to involvement (i.e., two-way communication between patients and HCPs by patient participation in safety improvement projects) to extensive partnership (i.e., patients work together with the HCPs to improve patient safety in the context of their own care). Moving from mere consultation to extensive partnership, each stage requires greater participation and cooperation from all parties engaged in the process [15].

In the Eastern Mediterranean Region (EMR) of the World Health Organization (WHO), lack of data on the quality and safety of healthcare remains a challenge. Nonetheless, one regional study revealed that up to 18% of hospital admissions might involve adverse events, of which 80% are deemed preventable [16]. Countries in the EMR have demonstrated a strong commitment to improving the safety of care. In 2005, they endorsed a resolution (EM/RC52/R.4) by the Regional Committee aimed at improving patient safety in the EMR. Since then, several endeavors have been undertaken to advocate for patient safety, raise awareness among health professionals and policymakers, and develop national and regional strategies to implement safe practices. One of the most important strategies is the Patient Safety Friendly Hospital Initiative (PSFHI), launched in the Region in 2011 to promote and encourage safe health practices in hospitals [17]. Patient and public involvement is a key domain of the PSFHI manual, which includes standards related to raising awareness of patients on their rights, empowering patients and their relatives in shared decision-making, gathering feedback from patients, addressing patient’s concerns and complaints, and involving the community in various patient safety activities [18].

Mapping existing literature on a given topic helps foster an understanding of the subject's academic development, identifies gaps in existing research, and potentially supports future research and practice directions. Despite the growing research on patient participation in patient safety globally [9, 11, 14], there is a lack of information on this topic among EMR member states. Therefore, we conducted a scoping review to examine the current state of the evidence on patient engagement in patient safety in the EMR. The review aimed to describe the breadth and depth of research regarding patients' engagement in safe care among EMR member states.

## Methods

### Approach

The scoping review was conducted to systematically describe the breadth and depth of the literature about patient engagement in safe care among member states of the EMR, which include Afghanistan, Bahrain, Djibouti, Egypt, Iran, Iraq, Jordan, Kuwait, Lebanon, Libya, Morocco, Occupied Palestinian Territory, Oman, Pakistan, Qatar, Saudi Arabia, Somalia, Sudan, Syrian Arab Republic, Tunisia, United Arab Emirates, and Yemen. Scoping reviews are increasingly utilized to identify gaps in evidence, guide research priorities, and identify implications for policy or practice [19]. A scoping review typically involves five main steps: scoping, searching, screening, data extraction, and data analysis [20]. Reporting of the scoping review was guided by the Preferred Reporting Items for Systematic Reviews and Meta-Analyses extension for Scoping Reviews (PRISMA-ScR) [21].

### Research questions

The research question for this study is: *“What is known regarding patient engagement in patient safety among member states of the EMR?*” We included studies focused on the following objectives: to investigate patients’ and families’ attitudes, perceptions, and experiences regarding their role in enhancing safety, and/or to investigate HCPs’ attitudes and beliefs about patient participation in patient safety practices, and/or to examine strategies and interventions for involving patients in safety activities within hospital settings.

### Search strategy

Following the guidelines for conducting systematic scoping reviews [22], a comprehensive literature search was conducted by a librarian using the electronic databases including PubMed, Medline, PsycINFO, CINAHL, Scopus, and ISI Web of Science with no date restriction in June 2023. Our search strategy consisted of combinations of three key blocks of terms related to “patient safety”(for example, medical error, adverse event, iatrogenic disease, infection control) “patient involvement” (for example, participat*, empower*, involv*, engag*), and “patients, families, and healthcare providers” (for example, patient, representative*, parent*, family, families, caregiver*, health provider, healthcare provider, clinician, physician, doctor, nurse, health professional, health worker). The search strategy for PubMed databases is provided in Additional file 1. The same strategy was adopted for other databases mentioned above, taking into account their different characteristics.

### Criteria for selection

We included empirical studies that directly explored patients’, families’ or HCPs’ attitudes, perceptions, and experiences related to patient engagement in safety activities. Additionally, studies were included if they explicitly or implicitly investigated the participation of patients, caregivers, or families in the design, delivery, and evaluation of the interventions aiming at promoting patient safety in inpatient settings. We included empirical qualitative, quantitative, and mixed-methods studies published in English in peer-reviewed journals. Additionally, the reference lists of all included studies were examined to identify additional relevant articles that may have been missed during the database search. The methodological quality of included studies was not assessed, as this is optional in scoping reviews [22, 23], and the purpose was only to describe the extent of existing research on the topic.

Titles and abstracts of the papers identified from the initial search were screened to determine if the full text should be retrieved. Two reviewers independently assessed the titles and abstracts against the inclusion criteria. Publications identified as potentially relevant were retrieved in full text and screened independently by three reviewers (ZA, SS, HN). Discrepancies regarding the inclusion of any publication were resolved through discussion and consensus among reviewers. Data extraction was conducted by two reviewers (SS, HN) using a structured abstraction form developed for this purpose. The form collected information on authors, year of publication, journal, country, research design, number and type of participants, barriers and facilitators to patient engagement, description of interventions, level of engagement, and main findings. The data extracted were cross-checked and verified by two other reviewers (HR, ZA).

The results were categorized based on the study's objectives and presented in a narrative form. We assessed the level of patient engagement in safety activities using a framework proposed by the NHS, which defines three levels of patient engagement: consultation (informing patients about patient safety and seeking patient feedback on safety issues), involvement (engaging patients in their care), and partnership (patients working together with HCPs as full team members to improve patient safety in the context of their own care) (partnership) [15].

## Results

**Fig. 1:**
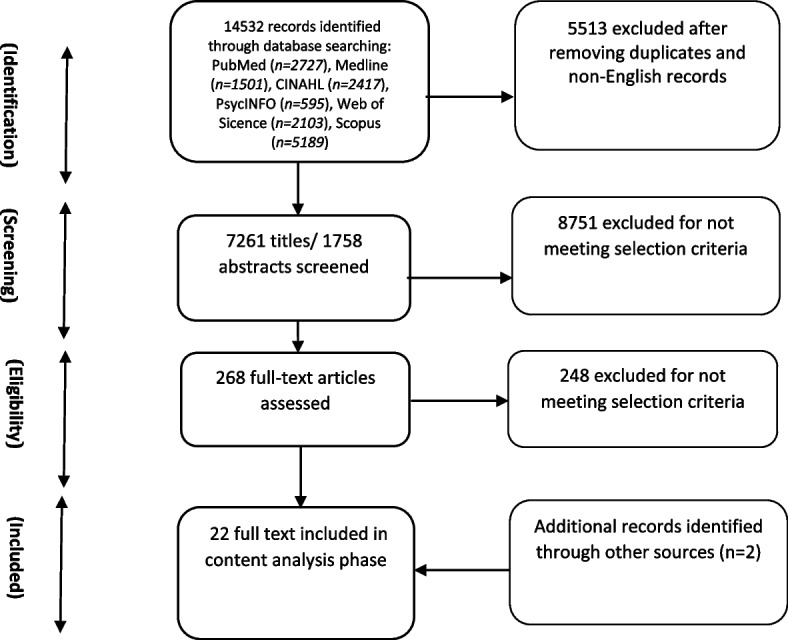
Information flow in scoping review

A total of 14,532 documents were initially identified from various databases: PubMed (*n* = 2,727), Scopus (*n* = 5,189), Medline (*n* = 1,501), PsycINFO (*n* = 595), Web of Science (2,103), and CINAHL (*n* = 2,417). After removing duplicates (*n* = 5,513), 9,019 unique records were evaluated based on title and abstract. Subsequently, 268 articles were assessed for full-text eligibility. Following the eligibility criteria, 248 articles were excluded. Two additional articles were identified through reference checking, resulting in a total of 22 studies published between 2011 and 2022 included in the current review (Fig. 1).

Among the included studies, nine were qualitative studies and 13 had quantitative designs, including quantitative surveys, quasi-experimental studies, and clinical trials. Sample sizes varied from 230 to 455 for quantitative and 19 to 94 for qualitative studies. Thirteen studies explored patients’, families’ or HCPs’ attitudes, perceptions, or experiences regarding preferences for patient engagement in patient safety, while nine publications reported actual patient engagement in safety activities. The publications included in the review were from Saudi Arabia, Iran, Pakistan, Jordan, and Lebanon. Detailed characteristics of the included studies are presented in Tables 1 and 2.

**Table 1:** Main characteristics of studies investigated patients’, families’ and providers’ perceptions and experiences of involvement in safe care

Study/ Country	Objective(s)	Design	Participants/ Settings	Main findings
Albalawi (2022)/ Saudi Arabia [24]	To explore healthcare professionals', patients' and families' perceptions and experiences of the patient safety culture and the impact of perceived barriers and facilitators on the implementation of a positive patient safety culture	Qualitative	35 HCPs, 8 patients, 4 family members at three hospitals in Madinah region	Participants believed that there was limited patient involvement in patient safety and a lack of sharing decision-making. Patients and families reported that there were limited processes and strategies that enabled them to take responsibility for their safety. HCPs believed that patients and families can contribute to patient safety by making sure they are responsible for their own care.
Alnasser et al. (2020)/Saudi Arabia [25]	To investigate the baseline status of patients’ awareness, knowledge, and attitudes to patient safety	Cross-sectional study (survey)	410 patients at King Khalid University Hospital, Riyadh	About 76.5% of patients would not ask their physician to wash their hands before the examination. Further, 58.6% said that they would be more willing to notify their physicians if they thought an error had occurred if were encouraged to do so.
Alshahrani et al. (2018)/ Saudi Arabia & Australia [26]	To explore the nature of relatives’ involvement in the care of patients in acute medical settings in Australia and Saudi Arabia and to explore the perceptions, attitudes and experiences of nurses	Qualitative	48 patients, 52 relatives, and 18 nurses at one hospital in Saudi Arabia	The results show that ambiguity regarding the role of relatives led to problems in patient care such as safety concerns and conflict with nurses. This could be addressed by the development and use of a set of principles or guidelines for nurses, relatives, and patients regarding relatives’ involvement.
Alwhaibi et al. (2020) /Saudi Arabia [27]	To explore the attitudes toward patient safety among medical students in Saudi Arabia	Cross-sectional (survey)	347 pharmacy students at four pharmacy colleges	A total of 53.5% of participants said that patients have an important role in preventing medical errors and 64.2% believed that encouraging patients to be more involved in their care can help to reduce the risk of medical errors occurring. Female students had a more positive attitude in the domains of patient safety including patient involvement in reducing error compared to their male counterparts.
Alyahya et al. (2018)/ Jordan[28]	To describe, examine, and evaluate the policies and procedures that are implemented to prevent and control hospital-acquired infections (HAIs) in the medical ICU in King Abdullah University Hospital	Qualitative	23 HCPs in medical ICU in King Abdullah University Hospital	Healthcare workers were aware but not fully engaged in preventing and controlling hospital-acquired infections; nevertheless, they presented themselves as knowledgeable. Staff recognized the importance of involving family members and visitors. However, they had serious concerns about open visitation.
Atoof et al. (2015)/ Iran [29]	To investigate the involvement of patients and public in the patient safety and treatment process in hospitals affiliated to Kashan University of Medical Sciences, Kashan, Iran, 2013	Cross-sectional (survey)	375 patients, 62 nurses and 18 chief managers in five hospitals in Kashan	Patient and public participation in Kashan hospitals were not at optimal levels. About 22% of patients were aware of their rights including being involved in decision-making related to the process of care. Only 20% of patients stated that hospitals received their feedback. More use of mass media and training programs was suggested to inform both the patients and the public on their rights and roles in improving the healthcare services.
Chegini et al. (2020) / Iran [30]	To investigate barriers to patient engagement in the delivery of safe hospital care	Qualitative	35 HCPs, managers, patient safety experts and researchers at Ministry of Health and medical universities in three provinces	Participants identified four main categories of barriers including 1) patient‐related barriers (e.g. low levels of health literacy, inadequate/ineffective education, patient unwillingness, and cultural barriers); 2) staff‐related barriers (e.g. the existence of negative attitudes towards engaging patients, ineffective communication, high workload); 3) Barriers created by limited resources and inadequate training in the workplace; 4) community‐related barriers (e.g. inadequate dissemination of information via the mass media and a lack of community‐based services).
Dehghan-Nayeri et al. (2015) / Iran [31]	To elicit experiences from nurses, families, and patients on the notion of family participation in the care of elderly patients in two general teaching hospitals in Iran	Qualitative	6 family members, 10 nurses and 5 elderly patients in two teaching hospitals in Tehran	Participants believed that family involvement in caregiving to elderly patients is important; yet nurses emphasized that participation should be based upon a planned and structured framework to ensure a safe and satisfying experience for patients, families, and health care team.
Farzi et al. (2017) / Iran [32]	To explore and describe the role of interactions among healthcare professionals regarding medication errors in intensive care units	Qualitative	19 HCPs (nurses, physicians, and clinical pharmacists) in 7 teaching hospitals in Isfahan	Weak Interaction of physicians and nurses with patients and families was recognized by HCPs as a potential source of medication errors. Active involvement and effective participation of patients and their families in the process of medication reconciliation is the key strategy to reduce prescribing errors, therefore, preventing any harm to the patient.
Novinmehr et al. (2019) / Iran [33]	To assess older adults’ self-advocacy regarding patient safety behavior and its relationship with some demographic characteristics	Cross-sectional (survey)	230 patients aged over 60 years in 5 public hospitals in Hamedan	The attitudes of the participants towards self-efficacy (participating in health care and becoming active in the treatment process) and their self-advocacy behaviors were at a medium level. A lower behavior subscale score was found in older adults with more advanced age and those living in rural areas.
Olfati et al. (2016) /Iran [34]	To identify the factors that affect patient involvement in safe delivery	Qualitative	63 patients in four maternity hospitals in Shahroud, Qazvin and Tehran	Patients expressed their desire to know more about the process of care. They believed that HCPs did not provide the required instructions. Prior experience of a patient safety incident can result in increased involvement of patients.
Sarkhosh et al. (2022) /Iran [35]	To qualitatively explore healthcare providers’ and managers’ perspectives on patient participation in patient safety processes	Qualitative	31 participants including HCPs, safety and quality officers, and hospital managers form teaching hospitals in Tehran	Participants believed that patients and their families can play an effective role in maintaining and improving patient safety through different roles. However, a variety of barriers were identified at patients, providers, and system levels hindering patient participation in delivering safe care.
Vaismoradi et al. (2011) /Iran [36]	To explore patients’ understandings and feelings of safety during hospitalization	Qualitative	19 patients in an urban teaching hospital	Supporting mechanisms designed to improve the feeling of safety in hospitalized patients should consider the patient as a whole and emphasize the humanistic aspect of patient care. Patients were found to lose confidence and avoid future contact and cooperation if healthcare providers avoided partnership or left concerns unresolved.

**Table 2:** Main characteristics of studies implemented interventions that engaged patients and families to promote safety

Study/ Country	Objective(s)	Type of study	Description of intervention	Settings	Engagement level*	Main findings (patient safety outcomes)
Al-Dorzi et al. (2014)/ Saudi Arabia [37]	To improve hand hygiene practices in a tertiary-care ICU	Quasi non-controlled study	A stepwise multifaceted approach was implemented in a tertiary-care ICU, which included hand hygiene education for staff, rotating residents, families, and visitors, workplace reminders, and active feedback	Intensive Care Department of King Abdulaziz Medical City-Riyadh, Saudi Arabia	Involvement	Hand hygiene compliance improved significantly from 64% to over 80%, and this improvement was sustained over several months.
Al Mutair et al. (2020)/ Saudi Arabia [38]	To assess the effectiveness of a developed quality improvement program in preventing Hospital-acquired pressure ulcers (HAPUs)	Quasi non-controlled study	The intervention focused on building a wound care team, providing education to hospital staff, patients, and their families, data monitoring, and follow-up visits after discharge	Qatif Central Hospital, a tertiary hospital with 360 beds located in the eastern province of Saudi Arabia	Involvement	The intervention effectively reduced the incidence of HAPUs from 0.20% in 2014 to 0.06% in 2018.
Awaji & Al-Surimi (2016)/ Saudi Arabia [39]	To improve hand hygiene compliance with a focus on the role of patients in promoting healthcare workers’ compliance with hand hygiene practices	Quasi non-controlled study	Several strategies including educating patients and encouraging them to ask their HCPs about hand hygiene and using reminders for health workers were implemented.	An oncology unit in a hospital in Saudi Arabia	Partnership	The initial findings show a 15% increase in compliance among HCPs with the interventions during the 10-day project testing. Additional data collection is needed to confirm sustained improvement over time.
Chegini et al. (2022)/ Iran [40]	To investigate the effects of educational interventions on patients’ self-efficacy and falls prevention knowledge	Quasi non-controlled study	Patients received an educational pamphlet on fall prevention. Patient knowledge and fall prevention self-efficacy were measured before and after the intervention.	A hospital in Iran	Involvement	The mean fall prevention knowledge score improved significantly from 47.8% to 68.3% following educational interventions, indicating an enhanced understanding of fall prevention among patients.
Karaoui et al. (2021)/Lebanon [41]	To assess the impact of pharmacist-conducted anticoagulation education and follow-up on bleeding and readmission rates	Randomized controlled trial	Participants were inpatients ≥ 18 years discharged on an oral anticoagulant for treatment. The control group (*n* = 100) received the standard nursing counseling while the intervention group (*n* = 100) additionally received pharmacy counseling. Outcomes were readmission rates, any bleeding event at day 3 and 30 post-discharge.	Lebanese American University Medical Center – Rizk Hospital (LAUMC-RH), a tertiary care teaching hospital in Beirut	Involvement	Although pharmacist intervention did not reduce readmission rates, patients counseled by pharmacists established better communication with HCPs, as evidenced by significantly more clinic visits and calls within 3 days.
Karimi et al. (2018)/ Iran [42]	To investigate the effect of home-based training on the incidence of bedsores in patients with stroke, during the year 2017	Randomized controlled trial	In this clinical trial, 70 family caregivers of stroke patients were randomly assigned to control (*n* = 35) and intervention groups (*n* = 35). The intervention group received caregiver education. After 12 weeks, both groups were assessed for bedsores incidence according to guidelines.	Neurology Ward of Ali Ebne Abitaleb Hospital, Zahedan, Iran	Involvement	After the intervention, the incidence of bedsores was 25.7% in the intervention group and 48.6% in the control group, showing a significant difference between the groups in bedsore frequency.
Mousavi et al. (2020) /Iran [43]	To investigate the impact of the “SPEAK UP” program on awareness about patient safety and evaluate their willingness to participate in safety activities	Randomized controlled trial	Patients were divided into two groups (control and experimental), each comprising 50 patients, and received an educational intervention to increase awareness of patient safety and participation.	Shafa Hospital, Babolsar, Iran	Consultation	The implementation of the "SPEAK UP" program significantly increased patients' awareness of participating in their own care and safety.
Noman et al. (2012)/ Pakistan [44]	To establish a comprehensive surveillance system involving infection control practitioners, surgeons, staff, and patients aimed at improving the post-discharge surveillance of surgical site infections (SSIs)	Quasi non-controlled study	A novel surveillance system as part of a comprehensive infection control program was developed. To improve detection rates, the patient was also given instructions on the signs and symptoms of SSIs.	A 200-bed dedicated cardiac care hospital, Pakistan	Involvement	By ensuring the active participation of all stakeholders including patients, the post-discharge surveillance improved, allowing for accurate assessment of SSI rates.
Ahmadi et al. (2022)/Iran [45]	To improve patient safety by promoting patient engagement within the local context of a maternity hospital by implementing best practice	Quasi non-controlled study	A set of educational interventions was performed on 46 patients and 46 HCPs to increase their knowledge on patient involvement in safety practices	Shahid-Beheshti maternity hospital, Maragheh, Iran	Consultation	The knowledge and practice of HCPs improved post-interventions. Patients also significantly improved in detecting and reporting clinical changes, communicating errors, and participating in safety initiatives after educational interventions.

^*^The NHS framework was used to determine the level of patient engagement, which defines three levels of patient engagement: consultation (informing and seeking feedback), involvement (engaging in care), and partnership (collaborating with healthcare professionals to improve safety) [15]

### Patients’ and families’ perceptions and experiences of involvement in safe care

We included eight studies that either directly assessed patients' attitudes towards involvement in safe care as an independent study or investigated patients' knowledge and attitudes towards patient safety, incorporating components on patients' knowledge and attitudes towards patient/family involvement [24–26, 29, 31, 33, 34, 36] (Table 1). Seven studies focused on understanding patients' attitudes and thoughts about their potential role in ensuring safety at the direct care level. One study explored patient attitudes towards involvement in safety activities along with other aspects of patient safety. Among these studies, three were quantitative cross-sectional studies, while the remaining five were qualitative.

The general findings suggest that patients reported limited involvement in patient safety initiatives and considered their role as passive [24–26, 29, 31, 33, 34, 36]. For instance, a qualitative study conducted in two hospitals in Saudi Arabia reported that patients experienced limited supportive processes and strategies in place to enable them to take an active role in their own safety [24]. In a survey conducted by Novinmehr et al. (2019) among elder inpatients in Iran, 41% of the patients reported being involved in the safety of their own care [33]. Additionally, according to patients' views, healthcare organizations and providers did not consider patient engagement in safety activities as a priority. For instance, in a study conducted in Iran, only 20% of patients stated that hospitals received their feedback [29]. Several studies suggested that the lack of patient engagement in patient safety reflected a broader cultural phenomenon where patients and their families did not actively participate in medical decision-making [24, 34].

Patient involvement was less likely for actions and behaviors that challenged and required questioning healthcare professionals. For instance, in a survey conducted in a hospital in Saudi Arabia, 76.5% of patients reported that they would not ask their physician to wash their hands before the examination [25]. Some studies noted that cultural and social norms prevalent in Middle Eastern countries contributed to patients feeling powerless to express dissatisfaction with healthcare systems or voice opinions or complaints [24, 31].

Some studies suggested that patients who were female, younger, had higher levels of education, and have experienced errors were more willing to participate in error-prevention strategies [33, 34]. The role of HCPs was recognized as crucial in empowering patients in the involvement process. A positive patient-provider relationship centered on trust, respect for the patients’ doubts, and listening to their questions and concerns was reported as a contributing factor in this process [25, 36]. In summary, the mapping of the literature highlighted gaps and limitations in our current understanding of patients’ perceptions and experiences of involvement in safe care, both in terms of the depth of the evidence and clarity of the concept.

There was limited investigation of family members’ perceptions and experiences related to their participation in patient care to ensure safe care as a primary focus (Table 1) [24, 26, 31]. Three studies investigated caregivers’ and families’ attitudes about taking an active role in ensuring safe care at the direct care level. Patients' family members believed that they could play an important role in ensuring safety and preventing harms, supporting patients by voicing concerns on their behalf, facilitating the continuity of patient care, and enhancing the patient–provider relationship [24, 31]. They asserted that the role of family members increases when patients are too ill, too old, or cognitively impaired [31].

Despite these benefits, family participation was a challenging task. Using a qualitative ethnographic approach, Alshahrani et al. (2018) investigated the extent of family members' participation in the care of patients in acute care settings in Australia and Saudi Arabia from the perspectives of nurses and relatives. In the Saudi Arabia setting, nurses reported feeling confused due to their dual role of caring for patients while also fulfilling organizational objectives that encourage partnerships with patients and their relatives. They asserted that the lack of policies and guidelines defining their roles and responsibilities in coordinating patients' and families’ involvement contributed to the role ambiguity [26]. In another qualitative study conducted by Dehghan-Nayery et al. (2015) in two general hospitals in Iran, the perspectives of patients, families, and HCPs towards family participation were investigated. Participants expressed positive attitudes towards involving family members in caregiving for elderly patients. However, they mentioned that the lack of policies and guidelines clearly outlining the roles and responsibilities of medical team members was a major barrier to patient involvement [31].

### HCPs' perceptions and preferences regarding patient involvement in safe care

Nine publications examined HCPs’ perceptions regarding patients’ systematic engagement in safety, either as their primary focus or as part of broader discussions on safety behaviors [24, 26–32, 35]. Among these, four studies investigated the attitudes of patients, relatives, and HCPs, while five studies focused solely on the attitudes of HCPs (Table 1). The HCPs included nurses, physicians, pharmacists, medical students, and administrators with varying years of experience in their profession and from different healthcare fields. The sample sizes varied from 10 to 38 for qualitative studies and 80 to 347 for surveys.

HCPs generally believed that patients have an important role in preventing medical errors, and active involvement of patients and families can represent an opportunity to reduce harms and risks. Participants mentioned several benefits for patient engagement, including better patient outcomes, reduced harms and increased safety, fewer complaints, and higher satisfaction [24, 27, 30, 35]. HCPs’ positive attitudes towards patient engagement in safety were identified as key to facilitating patient engagement in safety activities by several included studies [24, 26, 30]. However, a few included studies reported provider-related barriers to patient involvement in safety activities, including negative attitudes towards engagement, high workload and time constraints, lack of motivation and willingness, and lack of effective patient-provider communication [26, 30, 35]. Included studies emphasized that patient-provider interactions can facilitate or hinder the success of any efforts to improve safety [30]. The involvement of patients in their own care was considered as closely linked to the relationship established with health professionals [32, 35].

### Engaging patients and families in safety improvement interventions

Nine publications reported patients' and families’ participation in safety improvement interventions [37–45]. We categorized the included studies into two types: (1) independent projects aimed at directly promoting patient/family engagement in safety practices; or (2) patient safety improvement projects where patient/family engagement was a key component. Details of the interventions are summarized in Table 2.

Five quasi-experimental or randomized studies examined patient participation in safety improvement initiatives where patient involvement was not the primary focus. In a study conducted in a tertiary care ICU in Saudi Arabia, trained ICU staff audited the hand hygiene practices of HCPs and families using the WHO audit tool. Following the implementation of a stepwise multifaceted approach that included education, audit, and feedback, hand hygiene compliance significantly improved to 80%, and this improvement was sustained over several months [37]. Another study conducted in a hospital in Saudi Arabia reported active patient involvement in a wound care team aimed at reducing hospital-acquired pressure ulcers (HAPUs). The program focused on establishing a wound care team, providing education to HCPs, patients, and their families, as well as implementing surveillance and follow-up visits. The results demonstrated a significant reduction in the percentage of patients who developed pressure ulcers (PUs), decreasing from 0.20% to 0.06% over a period of 5 years [38]. Another study was a randomized, unmasked interventional trial conducted in a tertiary care teaching hospital in Lebanon. The study examined the effects of pharmacist-managed anticoagulation education and follow-up on bleeding and readmission rates among patients aged 18 years and older discharged on oral anticoagulants for treatment. The findings indicated that while the intervention did not lead to a reduction in bleeding or readmission rates, pharmacist education significantly improved patient-provider communication during the post-discharge period [41]. In another clinical trial conducted in Iran to investigate the effect of home-based education on the incidence of pressure ulcers in stroke patients, 70 family members were selected using convenience sampling and then randomly assigned to control and intervention groups. In the intervention group, educational sessions were conducted for family caregivers on stroke, pressure ulcers, and methods for preventing and treating pressure ulcers in stroke patients. The study reported a statistically significant difference in the incidence of pressure ulcers between the control and intervention groups [42]. In a study conducted in Pakistan, a comprehensive surveillance system involving HCPs and patients was implemented in a cardiac hospital to enhance the monitoring of surgical site infections post-discharge. Patients were educated about the signs and symptoms of surgical site infections and instructed to seek prompt assistance in the emergency room if any symptoms arose. The surveillance system successfully detected 22 infections out of 538 procedures, with 95% of these infections being identified during the post-discharge period [44]. All five studies evaluated the effectiveness of these strategies in reducing incidents, but none of them formally assessed patients' or families' experiences with the engagement activities.

Four out of nine studies described patient involvement in care as an institutional program designed to promote patient engagement in safe care. Three studies actively promoted engagement through educational strategies, such as learning sessions and training materials on patient safety, targeting both patients and HCPs. All three studies reported increased knowledge among patients and HCPs following these educational interventions [40, 43, 45]. One study conducted in an oncology unit at a hospital in Saudi Arabia, investigated the impact of patient involvement on promoting hand hygiene practices among HCPs. The study implemented several plan-do-study-act (PDSA) cycles, which were pilot-tested before full-scale implementation. Interventions included educational sessions aimed at empowering patients and improving HCPs' adherence to hand hygiene practices. The study reported that active patient involvement led to an increase in HCPs' compliance rate from 5 to 20% during the study period [39].

The included studies generally provided limited details about the involvement strategies, experiences of patients and caregivers with these strategies, and the factors that influenced their participation. Patient involvement in patient safety activities varied across a continuum, ranging from mere consultation to more active involvement and extensive partnership. Two studies reported consultation activities where patients and caregivers were educated about safety and asked for their feedback on safety incidents [43, 45]. Six studies focused on involvement activities, where patients and families served as members of improvement project teams or provided education to other patients and family members [37, 38, 40–42, 44]. Notably, one study went further by actively involving patients as partners in a quality improvement project aimed at enhancing hand hygiene compliance among HCPs [39].

### Facilitators and barriers to patient engagement in patient safety

Two studies specifically examined barriers and facilitators to patient engagement from the perspective of HCPs and managers [30, 35]. However, several other studies have reported additional factors that serve as barriers and facilitators to patient engagement in ensuring safety of care. These reported barriers and facilitators to patient engagement in patient safety can be categorized into three main categories, as shown in Table 3:

**Table 3:** Barriers to patient engagement reported by included studies

Category	Theme	Subthemes -reference(s)
Patient-related factors	Socio-demographic characteristics	• Age [33] • Language barriers [30, 31, 35] • Education [31, 33] • Physically or cognitively unable to participate [31, 35]
	Knowledge and skills	• Lack of patient awareness of healthcare risks [35] • Lack of knowledge of patient safety and terminology [33] • Lack of awareness of the patient's role in preventing harms and errors [24, 30, 34, 35, 43] • Low level of health literacy [30, 32, 35]
	Willingness and motivation	• Patient unwillingness due to different reasons including fear of reprisal, labeled as difficult patient [30, 35]
	Culture and values	• Feeling uncomfortable to challenge healthcare provider knowledge and authority [30, 31, 35, 39]
HCP-related factors	Knowledge	• Lack of knowledge among healthcare professionals on how to engage patients [26, 30, 35]
	Attitudes	• Negative attitudes and reluctance towards patient engagement [30, 35, 45] • Fear of legal laibilty [26, 35]
	Factors involving the relationship between patients and healthcare providers	• Poor interaction and ineffective communication between patients and HCPs [26, 30–32, 35, 45] • High workload and lack of time [31, 35, 38, 45]
Healthcare setting-related factors	Leadership and institutional support	• Lack of policies and guidance on the role of patients and how they should be involved [26, 31] • Lack of training/retraining programs for health professionals [27, 30, 35] • Lack of clarity in the roles and responsibilities [31, 35] • Lack of resources [28, 30, 35]
	Safety culture	• Lack of patient-centered approach [36]

#### Patient-related factors

Higher education and younger age were associated with a greater willingness to participate in error-reduction strategies [33, 34]. Patients’ illness-related factors, such as terminal illness, confusion, and general frailty, were identified as predictor factors for patient and their family involvement in the safety of their health care [31, 35]. In addition, language barriers hindered patient-provider communication, resulting in patients feeling reluctant or less able to actively participate [30, 31, 35]. One of the primary barriers preventing patients from participating in patient safety practices was their lack of knowledge and awareness about medical errors and patient safety [33]. Inadequate health literacy and poor knowledge were reported as major barriers to patient involvement in several studies [30, 34, 35, 42]. Raising public awareness, which will make patients more knowledgeable about patient safety and the possibility of medical errors, was identified as a facilitator to enhance patient involvement in safety activities [25, 29]. Clearly defined roles for patients, along with delivering education and training programs for both patients and HCPs on patient involvement, were also identified as facilitators to actively engage patients in safety efforts [26, 35]. Patients who believed that they were vulnerable to patient safety incidents were more willing to actively participate in error reduction strategies [34]. Patients' self-efficacy and self-care were recognized as predictors of their willingness to engage in patient safety activities [33].

Several factors were reported as negatively influencing patients' motivation to engage in their care, including fear of repercussion due to raising concerns [30, 35], reluctance to disturb busy HCPs by asking questions, and unwillingness to question or criticize HCPs’ behaviors and decisions [31, 35]. The latter barriers may be rooted in the Middle Eastern culture, where patients often view healthcare professionals as authorities, leading them to be unwilling to express concerns or complaints during their hospital stay [31].

#### HCP-related factors

The knowledge, beliefs, and attitudes of HCPs towards patients and their participation in treatment and safety issues were recognized as major factors influencing patient participation. Negative attitudes held by staff about how patients could contribute were cited as one of the main barriers toward patient involvement. The main obstacles were the hierarchical and paternalistic culture among HCPs and their unwillingness to abandon their traditional role and share their decision-making power [30, 35], even though they may not express it overtly [35]. Additionally, the fear of legal liability further contributed to HCPs' negative attitudes [26, 35].

The way in which HCPs interacted and communicated with patients influenced patient engagement in health care [36]. Patient participation was more likely to be achieved when healthcare professionals appreciate patients as knowledgeable partners in care and provide feedback to their concerns [36]. HCPs perceived patient and family involvement as a time-consuming and challenging task, particularly in the absence of clear rules and guidelines [26, 31, 35, 38, 45]. High workload prevented HCPs from effectively managing and coordinating patient and family participation in caregiving [26, 28, 31, 35].

#### Healthcare setting-related factors

Successful partnerships with patients to reduce errors and enhance safety were achieved when patient participation was encouraged by organizational values and directions. However, the lack of guidance and information on how patients should be involved, coupled with insufficient clarification regarding relevant HCPs’ legal and ethical responsibilities, posed barriers to patients' involvement in safety efforts [26, 35]. The use of appropriate mechanisms to receive patients' feedback, such as surveys and suggestion boxes, to integrate patient and family perspectives into daily activities was mentioned as a facilitator of patient engagement [29].

Lack of professional training and continuing education programs to train HCPs was identified as a barrier to meaningful patient participation [30, 35]. Some studies emphasized the importance of investing in HCPs training to promote attitudinal changes and thereby achieve better healthcare outcomes [26, 30, 35]. Several studies identified a lack of patient safety culture as a factor contributing to resistance to patient involvement initiatives at different levels [24, 30, 36]. Organizational culture was described in some studies as a critical factor influencing patient involvement. An organization with a positive culture was characterized by leaders who prioritize safety over productivity and financial gains, adopt processes and incentives to promote patient-centered communication, and provide adequate resources, structure, and accountability to facilitate patient involvement at all levels of the organization [24, 35].

## Discussion

### Summary of main aims and key findings

We found that patient and family engagement is still an emerging area in patient safety research in the EMR, with few published studies. This literature review identified limitations in both the depth of evidence and the clarity of concepts. Although there has been an increase in the number of quality and safety-related studies in the region in recent years [46], the topic of patient engagement in patient safety has received less attention.

We identified eight studies that investigated the views and experiences of patients and families regarding their participation and contribution to ensuring they receive safe care. Additionally, there were nine studies that investigated HCPs' attitudes and behaviors regarding patients’ involvement and contribution in patient safety initiatives. Nine studies described the successes and challenges of implementing patient safety interventions involving patients and their caregivers. Our results indicate the necessity for further exploration of various aspects of patient involvement in safety activities, considering the perspectives of both patients/families and HCPs. Nonetheless, the findings of these studies are worth considering.

### Comparison with the literature

Our review indicated that patient involvement in safety is influenced by a variety of factor associated with patients, HCPs, and organizational characteristics. Patient-related factors influencing their willingness to participate in their own healthcare process included patients’ acceptance of their new role in ensuring safe care, lack of medical knowledge, low confidence, presence of comorbidities, limited awareness of healthcare risks, reluctance to challenge or question HCPs’ knowledge and authority, low self-efficacy in preventing errors, fear of legal and technical implications when raising concerns, and various socio-demographic parameters. Our results are consistent with similar reviews examining patients' attitudes and willingness to participate in safety behaviors [11, 47–49]. While healthcare organizations cannot control certain patient-related barriers to patient participation, such as personal factors, they can address others by adopting appropriate actions. Patient empowerment plays a critical role in enhancing patient participation, particularly in error-reduction strategies [50]. Patients must have sufficient information and understanding about their health conditions, healthcare processes, and systems to enable them to be knowledgeable partners in decision-making about their own health [6]. Empowering patients can increase their awareness of errors associated with modern healthcare and their potential role in reducing and eliminating such errors [51]. EMR member states, like other countries worldwide, should intensify efforts to raise public awareness of patient safety issues. This increased awareness is critical for engaging patients and their caregivers in meaningful patient safety activities. This can be achieved through several approaches, including implementing targeted educational campaigns to inform the public about key patient safety topics, providing comprehensive training for HCPs on effective communication and patient safety practices, fostering community engagement through collaboration with local organizations and non-governmental organizations (NGOs), offering patient and family empowerment tools such as informational materials and resources to promote active participation in healthcare, and establishing robust data collection systems to monitor patient safety trends and outcomes within healthcare settings [52–54].

The evidence indicates that HCPs’ beliefs, attitudes, and behaviors have a substantial impact on patient engagement [55]. Our results suggest that HCPs generally have a positive attitude to engaging patients; however, the existing literature is insufficient to draw concrete conclusions. The results indicate that among HCPs, the acceptance and promotion of patient participation are negatively influenced by several factors, including hierarchical and paternalistic cultures that prioritize maintaining control, personal beliefs, fear of legal liability, lack of time, and inadequate training in patient-provider communication. Similar reviews in other countries have also reported these factors, indicating a widespread challenge in fostering patient participation within healthcare systems [56–58]. If healthcare organizations aim to promote meaningful patient involvement in patient safety efforts, then they must actively encourage and empower HCPs to support patient participation [57]. The knowledge and beliefs of healthcare professionals are significant determinants of patient involvement [48]. To achieve meaningful and effective patient engagement, healthcare systems should strive for a cultural shift from the traditional paternalistic approach in care delivery to fostering a collaborative partnership between patients and HCPs. This shift aims to support patients and enhance their capacities to become more informed, engaged, and proactive in their care [11].

Regarding institutional obstacles, one of the primary concerns highlighted by the included studies was the absence of a patient/people-centered approach that integrates patient and family perspectives and involvement at the point of care. The presence of an organizational culture that acknowledges the significance of patient involvement in ensuring safe care was identified as a crucial success factor for fostering such participation in the literature [14, 58]. Additionally, having leaders who focus on creating a transparent and receptive environment, along with implementing policies and mechanisms that promote patient-centered communication and shared decision-making among patients, their families, and HCPs, is recognized as essential for successful patient involvement initiatives [59].

In conclusion, promoting patient engagement necessitates the implementation of several strategies targeting patients, providers, and healthcare systems. It requires prioritizing safety at all levels of the healthcare system, ranging from direct care at the individual level to organizational governance, systems design, and policy-making [60]. A growing body of literature worldwide addresses the development and utilization of interventions to promote patient engagement in patient safety. However, mapping the literature within the EMR has revealed limitations in our current understanding of the topic and underscored the necessity for further research.

### Implications for future research and practice

There is limited research on the attitudes, perceptions, and experiences/preferences of patients, families, and HCPs regarding patient engagement across EMR countries. Studies have reported improvement initiatives without specifying how they engaged patients and families. Therefore, there is a significant need for empirical research to explore first, the feasibility and acceptability of patient participation in safety-related initiatives from the perspectives of patients, families, and HCPs, and second, whether such involvement contributes to improvements in safety. Understanding the challenges encountered by patients, families, and HCPs is essential for fostering meaningful patient engagement in safety-related behaviors across different contexts. By identifying barriers and challenges, targeted interventions can be designed and implemented for patients and providers. Future research should also examine the costs and benefits of patient involvement in safety improvement initiatives, assessing their effectiveness in reducing errors and harms. This approach aligns with the PSFHI, implemented by EMR hospitals since 2011, which provides clear standards for the role of patients and the public in enhancing healthcare safety [17].

The concept of patient engagement in patient safety spans a continuum from one-way information sharing to two-way collaboration and partnership between patients and providers. While adopting an in-depth participatory approach with extensive patient and family involvement may pose certain challenges for HCPs, several studies have reported benefits from this approach [10, 14]. Further efforts are needed to design and implement interventions that promote patient involvement to enhance patient safety in EMR countries. Future studies could focus on hospitals that have implemented the PSFHI framework to explore how patient participation in patient safety has been addressed and reinforced.

### Limitations

This review has several limitations that should be considered when interpreting and utilizing the findings. Firstly, we only included papers published in English, which may have led to the exclusion of relevant papers in other languages that could have provided further insights on the topic. Additionally, some of the reviewed papers did not specifically align with the objective of this scoping review, making it challenging to extract the required information. Moreover, due to the small number of studies, we did not exclude any based on quality. Lastly, this study specifically focused on patient and family participation in hospital settings and did not encompass studies from other settings. Given the importance of this subject, we recommend further studies in diverse settings to broaden our understanding. Nonetheless, this scoping review represents the first attempt to map the status of research on patient participation in patient safety initiatives in the region.

## Conclusion

Despite the international movement to increase patient involvement in safety, there is a lack of research evidence from the EMR on the acceptability to patients, families, and HCPs, as well as the potential impact of such involvement on enhancing safety. Available evidence suggests that patients are willing and capable of being involved in patient safety practices. There is a critical need to understand how patients, families, and caregivers can actively participate and contribute as knowledgeable partners in patient safety activities. Additional evidence is needed to understand the preferences and experiences of patients, caregivers, and HCPs regarding the involvement process, and whether such involvement leads to improved safety in practice. Future studies should aim to expand our understanding of which strategies work best in different contexts and their impact on patient safety. Particularly, there is a need for implementation studies that demonstrate how to effectively implement these concepts in clinical settings, given the diversity of the EMR countries.

### Supplementary Information

Supplementary Material 1.

## Data Availability

All data generated or analyzed during this study are included in this published article and its supplementary information files.

## Abbreviations

EMR: Eastern Mediterranean Region
HCP: Healthcare Provider
PRISMA-ScR: Preferred Reporting Items for Systematic Reviews and Meta-Analysis Extension for Scoping Review
PSFHI: Patient Safety Friendly Hospital Initiative
WHO: World Health Organization
