# Supplementary material for: Patient and family engagement in patient safety in the Eastern Mediterranean Region: a scoping review
Source: BMC Health Serv Res. 2024 Jun 25;24:765. doi: 10.1186/s12913-024-11198-3 (PMC11202252; doi:10.1186/s12913-024-11198-3)
Supplement: Supplementary file 1 — Supplementary Material 1. [file 12913_2024_11198_MOESM1_ESM.doc]

**Additional file 1. PubMed Search strategy (performed in 13 July 3023)**

#1. ("Patient Safety"[Mesh] OR "Risk Management"[Mesh:NoExp] OR "Safety Management"[Mesh:NoExp] OR "Medical Errors"[Mesh] OR "Patient Harm"[Mesh] OR "Iatrogenic Disease"[Mesh] OR "Long Term Adverse Effects"[Mesh] OR "Postoperative Complications"[Mesh:NoExp] OR "Health Services/adverse effects"[Mesh] OR "Hand Hygiene"[Mesh] OR "Cross Infection"[Mesh] OR "infection control"[Mesh:NoExp] OR "Accidental falls"[Mesh])= [715,550](https://pubmed.ncbi.nlm.nih.gov/?term="Patient+Safety"%5BMesh%5D+OR+"Risk+Management"%5BMesh%3ANoExp%5D+OR+"Safety+Management"%5BMesh%3ANoExp%5D+OR+"Medical+Errors"%5BMesh%5D+OR+"Patient+Harm"%5BMesh%5D+OR+"Iatrogenic+Disease"%5BMesh%5D+OR+"Long+Term+Adverse+Effects"%5BMesh%5D+OR+"Postoperative+Complications"%5BMesh%3ANoExp%5D+OR+"Health+Services%2Fadverse+effects"%5BMesh%5D+OR+"Hand+Hygiene"%5BMesh%5D+OR+"Cross+Infection"%5BMesh%5D+OR+"infection+control"%5BMesh%3ANoExp%5D+OR+"Accidental+falls"%5BMesh%5D&sort=relevance&size=200&ac=no)

#2. (safet* OR error* OR mistak* OR "risk management" OR "adverse event*" OR iatrogenic OR inciden* OR "sentinel event*" OR "near miss*" OR "adverse effect*" OR "patient harm*" OR "patient risk*" OR "infection control" OR "infection prevention" OR "Hand hygiene" OR "safe surgery"[Title/Abstract/Other term])= [2,423,919](https://pubmed.ncbi.nlm.nih.gov/?term=(safet*%5BOther+Term%5D+OR+error*%5BOther+Term%5D+OR+mistak*%5BOther+Term%5D+OR+"risk+management"%5BOther+Term%5D+OR+"adverse+event*"%5BOther+Term%5D+OR+iatrogenic%5BOther+Term%5D+OR+inciden*%5BOther+Term%5D+OR+"sentinel+event*"%5BOther+Term%5D+OR+"near+miss*"%5BOther+Term%5D+OR+"adverse+effect*"%5BOther+Term%5D+OR+"patient+harm*"%5BOther+Term%5D+OR+"patient+risk*"%5BOther+Term%5D+OR+"infection+control"%5BOther+Term%5D+OR+"infection+prevention"%5BOther+Term%5D+OR+"Hand+hygiene"%5BOther+Term%5D+OR+"safe+surgery"%5BOther+Term%5D)+OR+(safet*%5BTitle%2FAbstract%5D+OR+error*%5BTitle%2FAbstract%5D+OR+mistak*%5BTitle%2FAbstract%5D+OR+"risk+management"%5BTitle%2FAbstract%5D+OR+"adverse+event*"%5BTitle%2FAbstract%5D+OR+iatrogenic%5BTitle%2FAbstract%5D+OR+inciden*%5BTitle%2FAbstract%5D+OR+"sentinel+event*"%5BTitle%2FAbstract%5D+OR+"near+miss*"%5BTitle%2FAbstract%5D+OR+"adverse+effect*"%5BTitle%2FAbstract%5D+OR+"patient+harm*"%5BTitle%2FAbstract%5D+OR+"patient+risk*"%5BTitle%2FAbstract%5D+OR+"infection+control"%5BTitle%2FAbstract%5D+OR+"infection+prevention"%5BTitle%2FAbstract%5D+OR+"Hand+hygiene"%5BTitle%2FAbstract%5D+OR+"safe+surgery"%5BTitle%2FAbstract%5D)&sort=relevance&size=200&ac=no)

#3. ("fall injury" OR "fall injuries" OR "falls injury" OR "falls injuries" OR "fall risk" OR "fall risks" OR "falls risk" OR "falls risks" OR "fall prevention" OR "falls prevention" OR "fall patient" OR "fall patients" OR "falls patient" OR "falls patients")[Title/Abstract:~5]= [31,741](https://pubmed.ncbi.nlm.nih.gov/?term="fall+injury"%5BTitle%2FAbstract%3A~5%5D+OR+"fall+injuries"%5BTitle%2FAbstract%3A~5%5D+OR+"falls+injury"%5BTitle%2FAbstract%3A~5%5D+OR+"falls+injuries"%5BTitle%2FAbstract%3A~5%5D+OR+"fall+risk"%5BTitle%2FAbstract%3A~5%5D+OR+"fall+risks"%5BTitle%2FAbstract%3A~5%5D+OR+"falls+risk"%5BTitle%2FAbstract%3A~5%5D+OR+"falls+risks"%5BTitle%2FAbstract%3A~5%5D+OR+"fall+prevention"%5BTitle%2FAbstract%3A~5%5D+OR+"falls+prevention"%5BTitle%2FAbstract%3A~5%5D+OR+"fall+patient"%5BTitle%2FAbstract%3A~5%5D+OR+"fall+patients"%5BTitle%2FAbstract%3A~5%5D+OR+"falls+patient"%5BTitle%2FAbstract%3A~5%5D+OR+"falls+patients"%5BTitle%2FAbstract%3A~5%5D&sort=relevance&size=200&ac=no)

#4. ((fall AND injur*) OR (falls AND injur*) OR (fall AND risk*) OR (falls AND risk*) OR (fall AND prevention) OR (falls AND prevention) OR (fall AND patient*) OR (falls AND patient*))[Other term]= [3,259](https://pubmed.ncbi.nlm.nih.gov/?term=(fall%5BOther+Term%5D+AND+injur*%5BOther+Term%5D)+OR+(falls%5BOther+Term%5D+AND+injur*%5BOther+Term%5D)+OR+(fall%5BOther+Term%5D+AND+risk*%5BOther+Term%5D)+OR+(falls%5BOther+Term%5D+AND+risk*%5BOther+Term%5D)+OR+(fall%5BOther+Term%5D+AND+prevention%5BOther+Term%5D)+OR+(falls%5BOther+Term%5D+AND+prevention%5BOther+Term%5D)+OR+(fall%5BOther+Term%5D+AND+patient*%5BOther+Term%5D)+OR+(falls%5BOther+Term%5D+AND+patient*%5BOther+Term%5D)&sort=relevance&size=200&ac=no)

#5. "health infection" OR "health infections" OR "healthcare infection" OR "healthcare infections" OR "hospital infection" OR "hospital infections" [Title/Abstract:~1]= [20,624](https://pubmed.ncbi.nlm.nih.gov/?term="health+infection"%5BTitle%2FAbstract%3A~1%5D+OR+"health+infections"%5BTitle%2FAbstract%3A~1%5D+OR+"healthcare+infection"%5BTitle%2FAbstract%3A~1%5D+OR+"healthcare+infections"%5BTitle%2FAbstract%3A~1%5D+OR+"hospital+infection"%5BTitle%2FAbstract%3A~1%5D+OR+"hospital+infections"%5BTitle%2FAbstract%3A~1%5D&sort=relevance&size=200&ac=no)

#6. ("healthcare-associated infection" OR "healthcare-acquired infections" OR "hospital-acquired infection")[Other term]= [1,000](https://pubmed.ncbi.nlm.nih.gov/?term="healthcare-associated+infection"%5BOther+Term%5D+OR+"healthcare-acquired+infections"%5BOther+Term%5D+OR+"hospital-acquired+infection"%5BOther+Term%5D&sort=relevance&size=200&ac=no)

#7. (#1 OR #2 OR #3 OR #4 OR #5 OR #6)= [2,975,930](https://pubmed.ncbi.nlm.nih.gov/?term=longqueryafdc4220b49716ee2fb5&sort=relevance&size=200&ac=no)

#8. ("Caregivers"[Mesh] OR "Patients"[Mesh] OR "Family"[Mesh:NoExp] OR "Spouses"[Mesh] OR "Siblings"[Mesh] OR "Parents"[Mesh])= [356,804](https://pubmed.ncbi.nlm.nih.gov/?term="Caregivers"%5BMesh%5D+OR+"Patients"%5BMesh%5D+OR+"Family"%5BMesh%3ANoExp%5D+OR+"Spouses"%5BMesh%5D+OR+"Siblings"%5BMesh%5D+OR+"Parents"%5BMesh%5D&sort=relevance&size=200&ac=no)

#9. (patient* OR consumer* OR customer* OR citizen* OR public OR person* OR survivor* OR client* OR resident* OR user* OR communit* OR stakeholder* OR representative* OR parent* OR siblings OR family OR families OR caregiver* OR "care giver*" OR relative* OR carer*[Title/ Other Term])= [3,471,891](https://pubmed.ncbi.nlm.nih.gov/?term=(patient*%5BOther+Term%5D+OR+consumer*%5BOther+Term%5D+OR+customer*%5BOther+Term%5D+OR+citizen*%5BOther+Term%5D+OR+public%5BOther+Term%5D+OR+person*%5BOther+Term%5D+OR+survivor*%5BOther+Term%5D+OR+client*%5BOther+Term%5D+OR+resident*%5BOther+Term%5D+OR+user*%5BOther+Term%5D+OR+communit*%5BOther+Term%5D+OR+stakeholder*%5BOther+Term%5D+OR+representative*%5BOther+Term%5D+OR+parent*%5BOther+Term%5D+OR+siblings%5BOther+Term%5D+OR+family%5BOther+Term%5D+OR+families%5BOther+Term%5D+OR+caregiver*%5BOther+Term%5D+OR+"care+giver*"%5BOther+Term%5D+OR+relative*%5BOther+Term%5D+OR+carer*%5BOther+Term%5D)+OR+(patient*%5BTitle%5D+OR+consumer*%5BTitle%5D+OR+customer*%5BTitle%5D+OR+citizen*%5BTitle%5D+OR+public%5BTitle%5D+OR+person*%5BTitle%5D+OR+survivor*%5BTitle%5D+OR+client*%5BTitle%5D+OR+resident*%5BTitle%5D+OR+user*%5BTitle%5D+OR+communit*%5BTitle%5D+OR+stakeholder*%5BTitle%5D+OR+representative*%5BTitle%5D+OR+parent*%5BTitle%5D+OR+siblings%5BTitle%5D+OR+family%5BTitle%5D+OR+families%5BTitle%5D+OR+caregiver*%5BTitle%5D+OR+"care+giver*"%5BTitle%5D+OR+relative*%5BTitle%5D+OR+carer*%5BTitle%5D)&sort=relevance&size=200&ac=no)

#10. (#8 OR #9)= [3,651,071](https://pubmed.ncbi.nlm.nih.gov/?term=("Caregivers"%5BMesh%5D+OR+"Patients"%5BMesh%5D+OR+"Family"%5BMesh%3ANoExp%5D+OR+"Spouses"%5BMesh%5D+OR+"Siblings"%5BMesh%5D+OR+"Parents"%5BMesh%5D)+OR+((patient*%5BOther+Term%5D+OR+consumer*%5BOther+Term%5D+OR+customer*%5BOther+Term%5D+OR+citizen*%5BOther+Term%5D+OR+public%5BOther+Term%5D+OR+person*%5BOther+Term%5D+OR+survivor*%5BOther+Term%5D+OR+client*%5BOther+Term%5D+OR+resident*%5BOther+Term%5D+OR+user*%5BOther+Term%5D+OR+communit*%5BOther+Term%5D+OR+stakeholder*%5BOther+Term%5D+OR+representative*%5BOther+Term%5D+OR+parent*%5BOther+Term%5D+OR+siblings%5BOther+Term%5D+OR+family%5BOther+Term%5D+OR+families%5BOther+Term%5D+OR+caregiver*%5BOther+Term%5D+OR+"care+giver*"%5BOther+Term%5D+OR+relative*%5BOther+Term%5D+OR+carer*%5BOther+Term%5D)+OR+(patient*%5BTitle%5D+OR+consumer*%5BTitle%5D+OR+customer*%5BTitle%5D+OR+citizen*%5BTitle%5D+OR+public%5BTitle%5D+OR+person*%5BTitle%5D+OR+survivor*%5BTitle%5D+OR+client*%5BTitle%5D+OR+resident*%5BTitle%5D+OR+user*%5BTitle%5D+OR+communit*%5BTitle%5D+OR+stakeholder*%5BTitle%5D+OR+representative*%5BTitle%5D+OR+parent*%5BTitle%5D+OR+siblings%5BTitle%5D+OR+family%5BTitle%5D+OR+families%5BTitle%5D+OR+caregiver*%5BTitle%5D+OR+"care+giver*"%5BTitle%5D+OR+relative*%5BTitle%5D+OR+carer*%5BTitle%5D))&sort=relevance&size=200&ac=no)

#11. ("Health Personnel"[Mesh:NoExp] OR "Physicians"[Mesh] OR "Nurses"[Mesh] OR "Nursing Staff, Hospital"[Mesh] OR "Medical Staff, Hospital"[Mesh] OR "Patient Care Team"[Mesh])= [454,780](https://pubmed.ncbi.nlm.nih.gov/?term="Health+Personnel"%5BMesh%3ANoExp%5D+OR+"Physicians"%5BMesh%5D+OR+"Nurses"%5BMesh%5D+OR+"Nursing+Staff%2C+Hospital"%5BMesh%5D+OR+"Medical+Staff%2C+Hospital"%5BMesh%5D+OR+"Patient+Care+Team"%5BMesh%5D&sort=relevance&size=200&ac=no)

#12. (clinician* OR practitioner* OR physician* OR doctor* OR "health professional*" OR "health worker*" OR "health provider*" OR "healthcare provider*" OR "health care provider*" OR "care provider*" OR "care professional*" OR "health care professional*" OR "healthcare professional*" OR "health staff" OR "healthcare staff*" OR "health care staff*" OR nurs*[Title/ Other Term])= [563,986](https://pubmed.ncbi.nlm.nih.gov/?term=(clinician*%5BTitle%5D+OR+practitioner*%5BTitle%5D+OR+physician*%5BTitle%5D+OR+doctor*%5BTitle%5D+OR+"health+professional*"%5BTitle%5D+OR+"health+worker*"%5BTitle%5D+OR+"health+provider*"%5BTitle%5D+OR+"healthcare+provider*"%5BTitle%5D+OR+"health+care+provider*"%5BTitle%5D+OR+"care+provider*"%5BTitle%5D+OR+"care+professional*"%5BTitle%5D+OR+"health+care+professional*"%5BTitle%5D+OR+"healthcare+professional*"%5BTitle%5D+OR+"health+staff"%5BTitle%5D+OR+"healthcare+staff*"%5BTitle%5D+OR+"health+care+staff*"%5BTitle%5D+OR+nurs*%5BTitle%5D)+OR+(clinician*%5BOther+Term%5D+OR+practitioner*%5BOther+Term%5D+OR+physician*%5BOther+Term%5D+OR+doctor*%5BOther+Term%5D+OR+"health+professional*"%5BOther+Term%5D+OR+"health+worker*"%5BOther+Term%5D+OR+"health+provider*"%5BOther+Term%5D+OR+"healthcare+provider*"%5BOther+Term%5D+OR+"health+care+provider*"%5BOther+Term%5D+OR+"care+provider*"%5BOther+Term%5D+OR+"care+professional*"%5BOther+Term%5D+OR+"health+care+professional*"%5BOther+Term%5D+OR+"healthcare+professional*"%5BOther+Term%5D+OR+"health+staff"%5BOther+Term%5D+OR+"healthcare+staff*"%5BOther+Term%5D+OR+"health+care+staff*"%5BOther+Term%5D+OR+nurs*%5BOther+Term%5D)&sort=relevance&size=200&ac=no)

#13. (#11 OR #12)= [838,630](https://pubmed.ncbi.nlm.nih.gov/?term=("Health+Personnel"%5BMesh%3ANoExp%5D+OR+"Physicians"%5BMesh%5D+OR+"Nurses"%5BMesh%5D+OR+"Nursing+Staff%2C+Hospital"%5BMesh%5D+OR+"Medical+Staff%2C+Hospital"%5BMesh%5D+OR+"Patient+Care+Team"%5BMesh%5D)+OR+((clinician*%5BTitle%5D+OR+practitioner*%5BTitle%5D+OR+physician*%5BTitle%5D+OR+doctor*%5BTitle%5D+OR+"health+professional*"%5BTitle%5D+OR+"health+worker*"%5BTitle%5D+OR+"health+provider*"%5BTitle%5D+OR+"healthcare+provider*"%5BTitle%5D+OR+"health+care+provider*"%5BTitle%5D+OR+"care+provider*"%5BTitle%5D+OR+"care+professional*"%5BTitle%5D+OR+"health+care+professional*"%5BTitle%5D+OR+"healthcare+professional*"%5BTitle%5D+OR+"health+staff"%5BTitle%5D+OR+"healthcare+staff*"%5BTitle%5D+OR+"health+care+staff*"%5BTitle%5D+OR+nurs*%5BTitle%5D)+OR+(clinician*%5BOther+Term%5D+OR+practitioner*%5BOther+Term%5D+OR+physician*%5BOther+Term%5D+OR+doctor*%5BOther+Term%5D+OR+"health+professional*"%5BOther+Term%5D+OR+"health+worker*"%5BOther+Term%5D+OR+"health+provider*"%5BOther+Term%5D+OR+"healthcare+provider*"%5BOther+Term%5D+OR+"health+care+provider*"%5BOther+Term%5D+OR+"care+provider*"%5BOther+Term%5D+OR+"care+professional*"%5BOther+Term%5D+OR+"health+care+professional*"%5BOther+Term%5D+OR+"healthcare+professional*"%5BOther+Term%5D+OR+"health+staff"%5BOther+Term%5D+OR+"healthcare+staff*"%5BOther+Term%5D+OR+"health+care+staff*"%5BOther+Term%5D+OR+nurs*%5BOther+Term%5D))&sort=relevance&size=200&ac=no)

#14. (#10 OR #13)= [4,278,963](https://pubmed.ncbi.nlm.nih.gov/?term=longquerya6c0df9324a5843c5d85&sort=relevance&size=200&ac=no)

#15. ("Attitude to Health"[Mesh:NoExp] OR "Patient Participation"[Mesh] OR "Patient Advocacy"[Mesh] OR "Consumer Advocacy"[Mesh] OR "Stakeholder Participation"[Mesh] OR "Attitude of Health Personnel"[Mesh:NoExp] OR "Public Opinion"[Mesh] OR "Attitude"[Mesh:NoExp] OR "Patient Preference"[Mesh] OR "Refusal to Participate"[Mesh])= [338,750](https://pubmed.ncbi.nlm.nih.gov/?term="Attitude+to+Health"%5BMesh%3ANoExp%5D+OR+"Patient+Participation"%5BMesh%5D+OR+"Patient+Advocacy"%5BMesh%5D+OR+"Consumer+Advocacy"%5BMesh%5D+OR+"Stakeholder+Participation"%5BMesh%5D+OR+"Attitude+of+Health+Personnel"%5BMesh%3ANoExp%5D+OR+"Public+Opinion"%5BMesh%5D+OR+"Attitude"%5BMesh%3ANoExp%5D+OR+"Patient+Preference"%5BMesh%5D+OR+"Refusal+to+Participate"%5BMesh%5D&sort=relevance&size=200&ac=no)

#16. (participat* OR empower* OR activat* OR advoc* OR representative* OR involv* OR engag* OR collaborat* OR consult* OR contribut* OR willingness OR intention* OR sentiment* OR partner* OR opinion* OR role* OR awareness OR voice* OR experience* OR education OR "speak up" OR attitud* OR perspectiv* OR view*[Title/ Other Term])= [2,542,034](https://pubmed.ncbi.nlm.nih.gov/?term=(participat*%5BOther+Term%5D+OR+empower*%5BOther+Term%5D+OR+activat*%5BOther+Term%5D+OR+advoc*%5BOther+Term%5D+OR+representative*%5BOther+Term%5D+OR+involv*%5BOther+Term%5D+OR+engag*%5BOther+Term%5D+OR+collaborat*%5BOther+Term%5D+OR+consult*%5BOther+Term%5D+OR+contribut*%5BOther+Term%5D+OR+willingness%5BOther+Term%5D+OR+intention*%5BOther+Term%5D+OR+sentiment*%5BOther+Term%5D+OR+partner*%5BOther+Term%5D+OR+opinion*%5BOther+Term%5D+OR+role*%5BOther+Term%5D+OR+awareness%5BOther+Term%5D+OR+voice*%5BOther+Term%5D+OR+experience*%5BOther+Term%5D+OR+education%5BOther+Term%5D+OR+"speak+up"%5BOther+Term%5D+OR+attitud*%5BOther+Term%5D+OR+perspectiv*%5BOther+Term%5D+OR+view*%5BOther+Term%5D)+OR+(participat*%5BTitle%5D+OR+empower*%5BTitle%5D+OR+activat*%5BTitle%5D+OR+advoc*%5BTitle%5D+OR+representative*%5BTitle%5D+OR+involv*%5BTitle%5D+OR+engag*%5BTitle%5D+OR+collaborat*%5BTitle%5D+OR+consult*%5BTitle%5D+OR+contribut*%5BTitle%5D+OR+willingness%5BTitle%5D+OR+intention*%5BTitle%5D+OR+sentiment*%5BTitle%5D+OR+partner*%5BTitle%5D+OR+opinion*%5BTitle%5D+OR+role*%5BTitle%5D+OR+awareness%5BTitle%5D+OR+voice*%5BTitle%5D+OR+experience*%5BTitle%5D+OR+education%5BTitle%5D+OR+"speak+up"%5BTitle%5D+OR+attitud*%5BTitle%5D+OR+perspectiv*%5BTitle%5D+OR+view*%5BTitle%5D)&sort=relevance&size=200&ac=no)

#17. (#15 OR #16)= [2,767,784](https://pubmed.ncbi.nlm.nih.gov/?term=("Attitude+to+Health"%5BMesh%3ANoExp%5D+OR+"Patient+Participation"%5BMesh%5D+OR+"Patient+Advocacy"%5BMesh%5D+OR+"Consumer+Advocacy"%5BMesh%5D+OR+"Stakeholder+Participation"%5BMesh%5D+OR+"Attitude+of+Health+Personnel"%5BMesh%3ANoExp%5D+OR+"Public+Opinion"%5BMesh%5D+OR+"Attitude"%5BMesh%3ANoExp%5D+OR+"Patient+Preference"%5BMesh%5D+OR+"Refusal+to+Participate"%5BMesh%5D)+OR+((participat*%5BOther+Term%5D+OR+empower*%5BOther+Term%5D+OR+activat*%5BOther+Term%5D+OR+advoc*%5BOther+Term%5D+OR+representative*%5BOther+Term%5D+OR+involv*%5BOther+Term%5D+OR+engag*%5BOther+Term%5D+OR+collaborat*%5BOther+Term%5D+OR+consult*%5BOther+Term%5D+OR+contribut*%5BOther+Term%5D+OR+willingness%5BOther+Term%5D+OR+intention*%5BOther+Term%5D+OR+sentiment*%5BOther+Term%5D+OR+partner*%5BOther+Term%5D+OR+opinion*%5BOther+Term%5D+OR+role*%5BOther+Term%5D+OR+awareness%5BOther+Term%5D+OR+voice*%5BOther+Term%5D+OR+experience*%5BOther+Term%5D+OR+education%5BOther+Term%5D+OR+"speak+up"%5BOther+Term%5D+OR+attitud*%5BOther+Term%5D+OR+perspectiv*%5BOther+Term%5D+OR+view*%5BOther+Term%5D)+OR+(participat*%5BTitle%5D+OR+empower*%5BTitle%5D+OR+activat*%5BTitle%5D+OR+advoc*%5BTitle%5D+OR+representative*%5BTitle%5D+OR+involv*%5BTitle%5D+OR+engag*%5BTitle%5D+OR+collaborat*%5BTitle%5D+OR+consult*%5BTitle%5D+OR+contribut*%5BTitle%5D+OR+willingness%5BTitle%5D+OR+intention*%5BTitle%5D+OR+sentiment*%5BTitle%5D+OR+partner*%5BTitle%5D+OR+opinion*%5BTitle%5D+OR+role*%5BTitle%5D+OR+awareness%5BTitle%5D+OR+voice*%5BTitle%5D+OR+experience*%5BTitle%5D+OR+education%5BTitle%5D+OR+"speak+up"%5BTitle%5D+OR+attitud*%5BTitle%5D+OR+perspectiv*%5BTitle%5D+OR+view*%5BTitle%5D))&sort=relevance&size=200&ac=no)

#18. (#7 AND #14 AND #17)= [63,456](https://pubmed.ncbi.nlm.nih.gov/?term=longquerya1adbca6d3aaaad70371&sort=relevance&size=200&ac=no)

#19. (Afghan* OR Bahrain* OR Djibout* OR Egypt* OR Iran* OR Iraq* OR Jordan* OR Kuwait* OR Leban* OR Libya* OR Morocc* OR Palestin* OR Oman* OR Pakistan* OR Qatar* OR Saudi OR Somal* OR Sudan* OR Syria* OR Tunis* OR Emirate* OR Yemen* OR Gaza* OR "middle east" OR "Eastern Mediterranean") [Text Word/ Affiliation])= [840,077](https://pubmed.ncbi.nlm.nih.gov/?term=(Afghan*%5BAffiliation%5D+OR+Bahrain*%5BAffiliation%5D+OR+Djibout*%5BAffiliation%5D+OR+Egypt*%5BAffiliation%5D+OR+Iran*%5BAffiliation%5D+OR+Iraq*%5BAffiliation%5D+OR+Jordan*%5BAffiliation%5D+OR+Kuwait*%5BAffiliation%5D+OR+Leban*%5BAffiliation%5D+OR+Libya*%5BAffiliation%5D+OR+Morocc*%5BAffiliation%5D+OR+Palestin*%5BAffiliation%5D+OR+Oman*%5BAffiliation%5D+OR+Pakistan*%5BAffiliation%5D+OR+Qatar*%5BAffiliation%5D+OR+Saudi%5BAffiliation%5D+OR+Somal*%5BAffiliation%5D+OR+Sudan*%5BAffiliation%5D+OR+Syria*%5BAffiliation%5D+OR+Tunis*%5BAffiliation%5D+OR+Emirate*%5BAffiliation%5D+OR+Yemen*%5BAffiliation%5D+OR+Gaza*%5BAffiliation%5D+OR+"middle+east"%5BAffiliation%5D+OR+"Eastern+Mediterranean"%5BAffiliation%5D)+OR+(Afghan*%5BText+Word%5D+OR+Bahrain*%5BText+Word%5D+OR+Djibout*%5BText+Word%5D+OR+Egypt*%5BText+Word%5D+OR+Iran*%5BText+Word%5D+OR+Iraq*%5BText+Word%5D+OR+Jordan*%5BText+Word%5D+OR+Kuwait*%5BText+Word%5D+OR+Leban*%5BText+Word%5D+OR+Libya*%5BText+Word%5D+OR+Morocc*%5BText+Word%5D+OR+Palestin*%5BText+Word%5D+OR+Oman*%5BText+Word%5D+OR+Pakistan*%5BText+Word%5D+OR+Qatar*%5BText+Word%5D+OR+Saudi%5BText+Word%5D+OR+Somal*%5BText+Word%5D+OR+Sudan*%5BText+Word%5D+OR+Syria*%5BText+Word%5D+OR+Tunis*%5BText+Word%5D+OR+Emirate*%5BText+Word%5D+OR+Yemen*%5BText+Word%5D+OR+Gaza*%5BText+Word%5D+OR+"middle+east"%5BText+Word%5D+OR+"Eastern+Mediterranean"%5BText+Word%5D)&sort=relevance&size=200&ac=no)

#20. (#18 AND #19)= [2,727](https://pubmed.ncbi.nlm.nih.gov/?term=longquery51815214eff06c081660&sort=relevance&size=200&ac=no)
